# On the Physiological Modulation and Potential Mechanisms Underlying Parieto-Occipital Alpha Oscillations

**DOI:** 10.3389/fncom.2018.00023

**Published:** 2018-04-04

**Authors:** Diego Lozano-Soldevilla

**Affiliations:** Institut d'Investigacions Biomèdiques August Pi i Sunyer, Barcelona, Spain

**Keywords:** magnetoencephalography, electroencephalography, brain oscillations, pharmacology, cognitive processes

## Abstract

The parieto-occipital alpha (8–13 Hz) rhythm is by far the strongest spectral fingerprint in the human brain. Almost 90 years later, its physiological origin is still far from clear. In this Research Topic I review human pharmacological studies using electroencephalography (EEG) and magnetoencephalography (MEG) that investigated the physiological mechanisms behind posterior alpha. Based on results from classical and recent experimental studies, I find a wide spectrum of drugs that modulate parieto-occipital alpha power. Alpha frequency is rarely affected, but this might be due to the range of drug dosages employed. Animal and human pharmacological findings suggest that both GABA enhancers and NMDA blockers systematically decrease posterior alpha power. Surprisingly, most of the theoretical frameworks do not seem to embrace these empirical findings and the debate on the functional role of alpha oscillations has been polarized between the inhibition vs. active poles hypotheses. Here, I speculate that the functional role of alpha might depend on physiological excitation as much as on physiological inhibition. This is supported by animal and human pharmacological work showing that GABAergic, glutamatergic, cholinergic, and serotonergic receptors in the thalamus and the cortex play a key role in the regulation of alpha power and frequency. This myriad of physiological modulations fit with the view that the alpha rhythm is a complex rhythm with multiple sources supported by both thalamo-cortical and cortico-cortical loops. Finally, I briefly discuss how future research combining experimental measurements derived from theoretical predictions based of biophysically realistic computational models will be crucial to the reconciliation of these disparate findings.

## Introduction

The alpha rhythm is by far the most prominent spectral fingerprint of the human brain. Almost 90 years later, its mechanism and sources still remain elusive. As mysterious as its physiology, the discovery of the alpha rhythm was driven by Hans Berger's belief that electromagnetic brain activity could be the biological substrate behind telepathy (Millett, 2001). To investigate his hypotheses, he set in motion an ambitions human electrophysiology research program which led to the invention of the most employed non-invasive electrophysiological method—the electroencephalogram (EEG). It was the possibility to record brain activity through EEG that paved the way for the subsequent discovery of human brain rhythms. He reported “waves of first order”: the fascinating waxing and waning 10 Hz periodicity produced in relaxed but awake participants (Berger, 1929). Adrian and Mathews replicated and extended Berger's original findings. They showed, amongst other things, how water-beetles suppress their alpha amplitude in response to light, or how flicker stimulation can entrain the intrinsic alpha rhythm—thus making a case for its cortical origins (Adrian and Matthews, 1934).

Years later, human invasive EEG studies confirmed the existence of alpha oscillations in subcortical brain regions such as the thalamus (Gücer et al., 1978) and the pulvinar nuclei (Albe-Fessard et al., 1966; Albe-Fessard, 1973). In trying to disentangle the thalamo-cortical interactions, Lopes da Silva found the coherence between pulvinar and visual cortex to be much stronger than the coherence between the lateral geniculate nucleus (LGN) and the cortex (Lopes da Silva et al., 1973b). Interestingly, cortico-cortical coherence remained high after partializing the thalamic contribution (Lopes da Silva et al., 1973b), suggesting at least two independent sources. The cognitive relevance of the pulvinar-to-cortex circuitry was convincingly demonstrated 39 years later. Saalmann et al. (2012) revealed that the pulvinar plays a key role in setting the frequency of neuronal communication between V4 and the temporo-occipital area (TEO) to around 10 Hz when monkeys allocated their attention to a visual target.

Taking together, the neural circuitry behind posterior alpha rhythm seems rather complex, with thalamic and cortical sources contributing independently, and presumably influencing each other (Lopes da Silva, 1991). Indirectly, the complexity underlying the alpha rhythm can be grasped from the limited amount of *in-vitro* and *in-vivo* models (Silva et al., 1991; Hughes et al., 2004, 2011; Lörincz et al., 2008, 2009) relative to other rhythms whose neural circuitry essentially relies on local connectivity, such as beta and gamma (Traub and Miles, 1991; Traub et al., 1999; Traub and Whittington, 2010).

In this invited review, I will first discuss some methodological challenges common in pharmacological studies employing EEG and magnetoencephalography (MEG) and I will propose useful ways to approach them. Second, I will cover recent animal electrophysiology studies and EEG and MEG pharmacological challenges (pharmaco-MEEG) to illustrate how diverse neuromodulators can influence the posterior alpha rhythm. Regarding the pharmaco-MEEG evidence, I will focus on studies that employed drugs affecting GABA, glutamate, acetylcholine, and serotonin in healthy control participants during resting and task conditions. Third, I will discuss these empirical findings in relation to current theoretical frameworks to discuss how well the functional role of the alpha rhythm fits with its physiological modulations. Lastly, I will discuss the need to develop biophysically realistic computational models which can generate realistic predictions to guide the next generation of pharmaco-MEEG studies.

## Parieto-occipital alpha oscillations are modulated by a myriad of pharmacological agents

### GABAergic enhancement decreases parieto-occipital alpha power

The link between the alpha rhythm and physiological inhibition is long-established. During the 60's, Andersen and Andersson performed a series of pharmacological experiments in anesthetized cats to elucidate the physiological basis of the alpha rhythm. Employing barbiturates, potent GABAergic agonists, they were able to create an animal model that produced robust spindling activity with very similar frequency as the resting alpha. They claimed that the occipital resting alpha was mainly contributed by a thalamic pacemaker constituted by inhibitory neurons that project into the cortex (Andersen and Andersson, 1968). Years later, ground-breaking work by Fernando Lopes da Silva convincingly demonstrated that the proposed barbiturate-induced spindle activity and the classical alpha rhythm were two different physiological phenomena (Lopes da Silva et al., 1973a, 1980). Notably, the barbiturate-induced spindle activity was topographically more widespread than the posterior alpha, the wave duration was shorter and the thalamo-cortical coherence stronger (Lopes da Silva et al., 1973a). Mircea Steriade, a world leading authority on the cellular basis of brain rhythms, summarized the issue as follows:“*The temptation to understand the mechanisms of this rhythm at the cellular level by recording spindle oscillations under barbiturate anesthesia (Andersen and Andersson*, *1968**) is understandable, but alpha and spindle waves are quite different oscillations. Indeed, while the frequencies of these two rhythms may overlap, their origins and especially their behavioral context are dissimilar*” (Steriade, 1999). These findings relaxed the alpha pacemaker hypothesis, establishing the thalamus as a prominent alpha source (Lopes da Silva et al., 1973a,b, 1980) in addition to occipital sources (Lopes da Silva and Storm Van Leeuwen, 1977).

Pharmaco-MEEG has employed a variety of anesthetics and barbiturates known to potentiate the effect of GABA to study its effect on alpha oscillations. Propofol, for example, is a popular anesthetic that binds to GABA_A_ receptors producing a strong hyperpolarization as a consequence of the opening of chloride channel in pyramidal neurons (Bai et al., 1999). This hyperpolarization led to a loss of consciousness, which is characterized by a prominent alpha power increase in frontal sensors with a concurrent posterior alpha power decrease (Ching et al., 2010; Cimenser et al., 2011; Supp et al., 2011; Purdon et al., 2013), a process coined as “anteriorization” (Feshchenko et al., 2004). Other drugs, such the benzodiazepines, are not agonists but allosteric modulators: they need the presence of GABA neurotransmitter in the synaptic cleft to produce the hyperpolarization. Relative to anesthetics and barbiturates, benzodiazepines produce milder sedative effects and have lower risk of over dosage. They bind to GABA_A_ receptors and enhance chloride conductance by increasing its channel-opening frequency (Riss et al., 2008), as opposed to maintaining the channels open (as anesthetics and barbiturates do). Interestingly, a large variety of benzodiazepine type drugs using a wide range of dosages have been shown to produce a consistent decrease in parieto-occipital alpha power during resting state (Fink et al., 1976; Bond and Lader, 1982; Golombok and Lader, 1984; Koopmans et al., 1988; Van Steveninck et al., 1993; Feshchenko et al., 1997; Liley et al., 2003; Boeijinga et al., 2004; Fingelkurts et al., 2004; Schreckenberger et al., 2004; Connemann et al., 2005; Ahveninen et al., 2007; Yoto et al., 2012; Alonso et al., 2015; Nutt et al., 2015; Lozano-Soldevilla et al., 2016) and task conditions (van Leeuwen et al., 1995; Gevins et al., 2002; Muñoz-Torres et al., 2011; Saxena et al., 2013; Lozano-Soldevilla et al., 2014). Reports of benzodiazepine studies finding posterior alpha power increases (Tran et al., 2004; Nikulin et al., 2005; Hall et al., 2010; Nutt et al., 2015), frequency modulations (Liley et al., 2003), or just null results (Urata et al., 1996; Jensen et al., 2005; Muthukumaraswamy et al., 2013b; Campbell et al., 2014) are less common within the literature. In conclusion, pharmacologically increasing GABA efficacy leads to a decrease in power of the classical alpha—but, what is the underlying mechanism?

### Are posterior alpha oscillations implemented by physiological inhibition, excitation, or a balance between the two?

Current theoretical frameworks assign alpha oscillations a *functional inhibitory* role. In this case, alpha power should exert *functional inhibition* on those brain regions, time periods or cognitive representations that need to be suppressed to optimally perform the task at hand (Klimesch et al., 2007; Jensen and Mazaheri, 2010; Mazaheri and Jensen, 2010; Foxe and Snyder, 2011; Mathewson et al., 2011; Weisz et al., 2011; Jensen et al., 2012, 2014; Klimesch, 2012; Womelsdorf et al., 2014). At the other extreme of the continuum, numerous studies have found that alpha performs an active role in numerous cognitive operations (Palva and Palva, 2007, 2011; Womelsdorf et al., 2014). These frameworks demonstrate a remarkable and parsimonious link between oscillatory activity and behavior. Through them, one can explain a myriad of electrophysiological, cognitive, and behavioral findings obtained in a large variety of tasks and sensory modalities. Key to the present discussion, some frameworks assume that the *functional inhibition* reflected in the alpha band is implemented through *physiological inhibition* (Klimesch et al., 2007; Jensen and Mazaheri, 2010; Mazaheri and Jensen, 2010; Jensen et al., 2012, 2014). More specifically, alpha oscillations could represent physiological inhibition generated by GABAergic interneurons and they could tax neuronal processing indexed by neuronal firing. This inhibition reflected in the alpha band has been modeled as a thalamic drive whose GABA conductance changes sinusoidally at 10 Hz (Gips et al., 2016). Alpha phase could play a fundamental role in segmenting visual information as a function of neuronal excitability: with more excitable cells firing earlier within the alpha cycle as this would allow them to code more salient stimuli (Jensen et al., 2012, 2014). See (Gips et al., 2016) for a computation implementation of the idea. Thereby, *functional inhibition* could be a consequence of physiological “pulsed” inhibition produced by GABAergic feedback carried by interneurons within the cortex or the thalamus (Jensen and Mazaheri, 2010; Mazaheri and Jensen, 2010). Other recent frameworks, although highlighting the inhibitory role of alpha, widen the physiological hypothesis stressing the importance of the excitatory and inhibitory balance to control alpha amplitude and frequency (Klimesch, 2012; Himmelstoss et al., 2015). Other accounts stress that the crucial characteristic of alpha function is that it blocks the communication of local circuits due to its relatively slow period which precludes the action of fast inhibition (Fries, 2015). Finally, Womelsdorf et al. (2014) recent framework proposes the existence of two dynamical motifs that could account for the inhibitory and the active views of alpha. A motif is defined as a neural network composed of different cell types that produce different rhythms which perform specific neuronal computations. They propose a thalamic motif that amplifies that transmission of attended sensory information (active role), and a cortical motif in L6 that processes irrelevant information and is manifested due to the lack of thalamic excitatory input (inhibitory role).

There are indeed *in-vivo* animal models showing the importance of inhibitory interneurons in the modulation of ~10 Hz rhythm in the somatosensory cortex and LGN (Fanselow and Nicolelis, 1999; Lörincz et al., 2009). In conclusion, if one follows the “*alpha power reflects functional inhibition”* principle, increasing the level of inhibition should lead to an increase of alpha power. The studies reviewed here indicate that pharmacological GABAergic enhancement decreases (rather than increases) the energy of the posterior alpha oscillations. This observation is not new and has indeed been acknowledged in the past:“*Finally, we should also emphasize that one of the most straight forward tests of our hypothesis apparently leads to inconsistent results. The application of benzodiazepines (a GABA agonist) does not lead to increased alpha activity as one would expect if alpha is related to inhibitory activity but instead to a decrease in alpha*” (Klimesch et al., 2007).

Most of the studies that used benzodiazepines and anesthetics recorded electrophysiological activity under resting state conditions. One may argue that the GABAergic modulations producing functional inhibition reflected within the alpha band could only be relevant (or measured) when the task at hand requires a strong top-down drive. Under these conditions, alpha power modulations have been shown to impact behavior in a variety of task and sensory modalities (Klimesch et al., 2007; Jensen and Mazaheri, 2010; Mazaheri and Jensen, 2010; Foxe and Snyder, 2011; Mathewson et al., 2011; Jensen et al., 2012, 2014; Klimesch, 2012).

**Figure 1:**
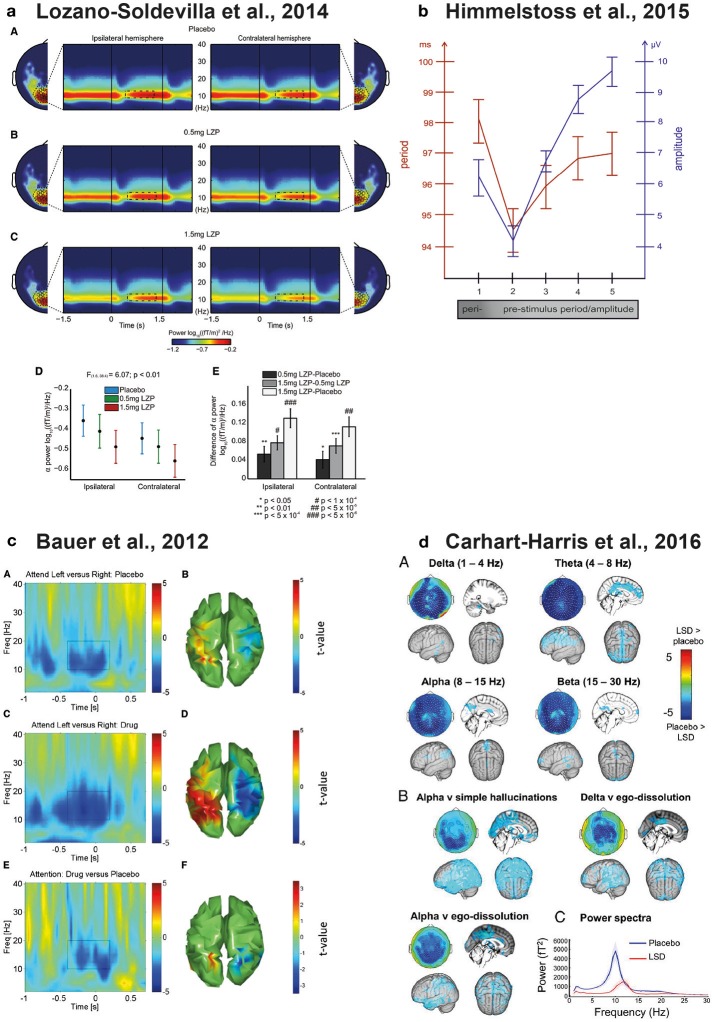
Physiological modulations of parieto-occipital alpha oscillations. **(a)** Lorazepam (GABAergic enhancer) parametrically reduces both tonic and attentional alpha power modulations during the delay interval of a visuo-spatial working memory task. The figure shows the topographic distribution of alpha power and the time and frequency dynamics of ipsilateral and contralateral sensors of interest under Placebo **(A)** 0.5 mg **(B)** and **(C)** 1.5 mg of Lorazepam. **(D,E)** show the delay-specific interaction between drug dosage and sensors meaning that Lorazepam caused a strong reduction in the ipsilateral relative to the contralateral sensors. Reproduced from Lozano-Soldevilla et al. (2014) with the permission from Elsevier. **(b)** Relationship between posterior alpha frequency and EEG amplitude. The x-axis represents the alpha cycles preceding the single trial P1 evoked potential produced in a semantic-judgement task. The left y-axis represents the period of the alpha band (red) and the right y-axis represents the amplitude of the EEG measured at the time point of the positive alpha peak (blue). The EEG raw amplitude correlates positively with alpha frequency. Reproduced from Himmelstoss et al. (2015) with the permission from Frontiers S.A. **(c)** Physostigmine (cholinergic enhancer) increases the attentional alpha power modulations in parieto-occipital cortices relative to placebo during the cue interval of a visuo-spatial attention task. **(A)** Time and frequency representation of the left minus right attention contrast during the placebo session of significant parieto-occipital voxels **(B)**. Physostigmine attention contrast and interaction are represented in **(C,D)** and **(E,F)**, respectively. Reproduced from Bauer et al. (2012) with the permission from Elsevier. **(d)** LSD (serotonergic enhancer) reduces posterior alpha power during resting state conditions **(A)**. The LSD-induced alpha power reduction correlates with increase of simple hallucination and ego-dissolution ratings **(B)**. LSD produced a robust increase of posterior alpha peak frequency **(C)**. Reproduced from Carhart-Harris et al. (2016) with the permission from Proceedings of the National Academy of Sciences of the United States of America.

Lozano-Soldevilla et al. (2014) designed a pharmaco-MEG study in humans to test this possibility. We combined MEG recordings testing two different dosages of lorazepam (0.5, 1.5 mg), a benzodiazepine that increases GABA efficacy. Participants performed a visuo-spatial working memory task where they had to allocate their locus of attention to a cued visual hemifield (1.5 s). After, a brief presentation (0.1 s) of a sample stimulus of colored squares, participants had to memorize the color of the cued items while trying to ignore the non-cued items. After a 1.5 s delay, a second array appeared and participants decided whether the color of the items between the sample and the probe matched or not. This task is ideal to test the *functional inhibition* role of alpha during the allocation of attentional (cue period), memory maintenance and distractor suppression (delay period). Previous studies have repeatedly demonstrated that alpha power can be used as an electrophysiological proxy for the allocation of attention (Foxe et al., 1998; Worden et al., 2000; Thut et al., 2006; Händel et al., 2010; Foxe and Snyder, 2011). The increase of GABA efficacy leads two main predictions. First, if alpha is exerting its *functional inhibition* role through *physiological inhibition*, GABAergic enhancement should increase tonic alpha power. Second, this alpha power attentional modulations should increase parametrically with drug dosage. We found robust evidence in the opposite direction for both predictions. First, lorazepam strongly decreased both tonic alpha power and attentional power modulations (Lozano-Soldevilla et al., 2014). Furthermore, we obtained an interaction between hemisphere and drug dosage specific to the delay interval. The interaction revealed that lorazepam caused an increased reduction in alpha power measured by the ipsilateral sensors relative to the contralateral sensors Figure 1a. What is the mechanism explaining the alpha power decrease with GABAergic enhancement? One possibility could be that the physiological bases of classical alpha oscillations rely on *physiological excitation* to a similar (or stronger) degree as *physiological inhibition*. Several lines of evidence support this speculation. *In-vivo* animal models have shown that pyramidal cells in the cortex have sufficient biophysical properties to produce alpha rhythm on their own (Steriade et al., 1990; Lopes da Silva, 1991; Silva et al., 1991; Castro-Alamancos and Connors, 1996; Flint and Connors, 1996). It is known that benzodiazepines decrease the number of spikes per burst, leaving the burst rate unaffected (Antkowiak, 1999). This finding could explain why benzodiazepines decrease alpha power without affecting its peak frequency.

It is interesting to note that very often the power dynamics underlying alpha and gamma oscillations tend to show opposite behavior. Regarding power modulations, while pharmacological GABAergic enhancement decreases alpha power, gamma power shows an increase (Oke et al., 2010; Xing et al., 2012a; Saxena et al., 2013; Campbell et al., 2014; Lozano-Soldevilla et al., 2014; Magazzini et al., 2016) but see (Muthukumaraswamy et al., 2013b,c). Stimulus-induced gamma power increase is often accompanied by alpha power decrease and this power asymmetry reverses in spontaneous conditions (Fries et al., 2001; Hoogenboom et al., 2006). A computational work proposes that this negative power relationship between high and low frequencies might be due to the ratio of the AMPA and NMDA conductances (Buehlmann and Deco, 2008; Deco and Thiele, 2009). Gamma oscillations seem to be located in more superficial layers while alpha is found to be stronger in infragranular layers (Bollimunta et al., 2008; Buffalo et al., 2011; Spaak et al., 2012; Xing et al., 2012b; van Kerkoerle et al., 2014; Bastos et al., 2015; Michalareas et al., 2016) but see (Haegens et al., 2015). In conclusion, alpha and gamma rhythms seem to rely on complementary physiological mechanisms.

Another possibility would be that the alpha rhythm relies on the fine-grained excitation and inhibition (E/I) balance (Klimesch, 2012). The E/I balance has been intensively studied for faster rhythms during both spontaneous and stimulus-evoked conditions (Anderson et al., 2000; Okun and Lampl, 2008). For example, there is compelling evidence showing that the instantaneous E/I balance determines peak gamma frequency (Atallah and Scanziani, 2009). Similarly, recording EEG in a semantic judgement task, (Himmelstoss et al., 2015) showed that alpha frequency and amplitude covary: alpha period lengthened during high amplitude time windows Figure 1b. If alpha amplitude is interpreted as instantaneous inhibition, the period of alpha must be slow. These results resemble the pyramidal-interneuron network gamma (PING) model. In this model, the minimum frequency is governed by the inhibitory time constant that prevents principal cells from firing (Tiesinga and Sejnowski, 2009). Unfortunately, these type of experimental models do not produce oscillations below 20 Hz (Traub et al., 1996).

Overall, these experimental and modeling results indicate that higher and lower frequencies are generated in different paths of the brain hierarchy: while gamma oscillations could be functionally associated with feedforward communication, alpha oscillations could be involved in feedback communication. Combining these findings, if it is true that alpha and gamma rhythms show different and often opposite neurophysiological behavior, it would not be naive to assume that *physiological excitation* is a key mechanism by which alpha oscillations exert their *functional inhibition*. In line with this hypothesis, blocking excitation by NMDA antagonists reduces alpha power in humans (Muthukumaraswamy et al., 2015) and monkeys (van Kerkoerle et al., 2014). The hypothetical mechanism by which this *physiological excitation* inhibits a brain area is, to the best of the author's knowledge, unknown.

### Glutamate receptors can shape alpha power and frequency

Glutamate is the principal excitatory neurotransmitter in the nervous system and it produces EPSPs when binding to NMDA or AMPA receptors. *In-vitro* and *in-vivo* animal models speak in favor of the potential role of glutamate in alpha generation. Silva et al. (1991) found that the intrinsic membrane properties of pyramidal neurons in somatosensory cortex layer V were both necessary and sufficient to fire periodically between 5 and 12 Hz. They showed that Na and NMDA were essential ingredients to produced synchronized oscillations. More recently, Hughes et al. (2004) convincingly demonstrated that metabotropic glutamate receptor mGluR1a activation in LGN increased the frequency and reduced the amplitude of neuronal oscillations between 2 and 13 Hz. Lörincz et al. (2009) demonstrated “*unequivocally that the temporal framing of relay-mode TC neuron activity results from phasic inhibition from LGN interneurons. However, a crucial component in ultimately bringing about this temporal framing is the rhythmic synaptic excitation of interneurons, because without this excitation these cells do not generate action potentials and the phasic inhibition of relay-mode TC neurons does not occur.”* Finally, a recent ground-breaking monkey study has found that both AMPA and NMDA blockers consistently suppressed alpha power in V1. Interestingly, NMDA blockers, but not AMPA, increased stimulus-induced gamma power within the same brain region and time-period (van Kerkoerle et al., 2014). This pharmacological dissociation was in line with the laminar findings, strengthening the argument that the physiological mechanisms behind alpha and gamma are complementary.

Under resting state conditions, recent pharmaco-MEEG studies found a decrease in parieto-occipital alpha power (Muthukumaraswamy et al., 2015; Rivolta et al., 2015; de la Salle et al., 2016) and frequency (Knott et al., 2006) using sub-anesthetic doses of ketamine. Surprisingly, the same ketamine and perampanel (AMPA blocker) produced no apparent effect—either in the posterior alpha power, or the frequency in visual stimulation tasks (Shaw et al., 2015; Muthukumaraswamy et al., 2016). Recent studies using ketamine in dosages that induce general anesthesia (loss of consciousness) show similar but stronger alpha modulations (Akeju et al., 2016; Vlisides et al., 2017). All in all, both animal and human experiments agree on the important role of glutamate neurotransmission modulating alpha power and frequency.

### Acetylcholine enhancement mostly increases posterior alpha power and frequency

Acetylcholine is an organic chemical that can function as both a neurotransmitter and as neuromodulator. It is generated in the pedunculopontine nucleus and in the basal nucleus of Meynert, projecting to various locations in the cortex, hippocampus, and amygdala. Due to its anatomical projections, acetylcholine is a key player during attentive, learning, and memory processes (Thiele, 2013).

It has been reported that while muscarinic blockers like scopolamine diminish the resting parieto-occipital alpha oscillations (Sannita et al., 1987; Sloan et al., 1992; Ebert et al., 1998, 2001; Kikuchi et al., 1999; Osipova et al., 2003; Ramos Reis et al., 2013), nicotinic administration produces the reverse effect (Gilbert et al., 2000; Knott et al., 2000, 2012; Walker et al., 2001; Bowers et al., 2015; Smith et al., 2016) but see (Knott and Fisher, 2007; Balsters et al., 2011; Fisher et al., 2012b). Acetylcholine enhancers tend to increase posterior alpha power during visuospatial attentional modulations (Bauer et al., 2012) Figure 1c and frontal alpha during working memory tasks (Fisher et al., 2012a, 2013) but see (Eckart et al., 2016). Other studies using visual stimulation paradigms reported posterior alpha power increase using mild nicotine dosages (Thompson et al., 2000) while higher dosages caused a decrease (Beaver et al., 2011; Beer et al., 2013). Interestingly, some studies have also found that enhancing cholinergic efficacy leads to a widespread scalp alpha period acceleration (Foulds et al., 1994; Knott et al., 1998, 2006; Domino et al., 2009; Bowers et al., 2015). How can acetylcholine modulate alpha power and frequency? A series of animal models revealed that cholinergic muscarinic receptors (mAChR) play a critical role in inducing intrinsic HT burst-firing in a subset of TC cells (Lörincz et al., 2008). Strikingly, some TC cells with HT bursting activity exhibited a phase-shifting in their spiking output. The phase-shifting varied from a predominantly in-phase relationship with the negative peak of the LFP to an anti-phase relationship. These phase-shifts occurred spontaneously and were determined by a combination of factors such as network input and its intrinsic properties or the polarization of cell-membranes (Lörincz et al., 2008).

### Serotonin (5-HT) can either enhance or suppress posterior alpha power

The Raphe nuclei is the main serotonin generator responsible for releasing the neurotransmitter to the vast majority of the brain. Regarding its cognitive functions, the prominent innervations between Raphe nuclei and prefrontal cortex suggest a major role in learning, working memory, and cognitive control (Puig and Gulledge, 2011).

A variety of serotonergic enhancers (Venlafaxine, Tianeptine, Ecstasy, Ayahuasca, Psylocibin, LSD) seem to decrease posterior alpha power during both resting (Saletu et al., 1986, 1992; Riba et al., 2002, 2004; Muthukumaraswamy et al., 2013a; Kometer et al., 2015; Schenberg et al., 2015; Carhart-Harris et al., 2016; Valle et al., 2016) but see (Link et al., 1991; Saletu et al., 1996; Knott et al., 2012) and task conditions (Kometer et al., 2013; Muthukumaraswamy et al., 2013a). The serotonergic system has been proposed to play a major role in the generation of visual hallucinations. Substances such as LSD, psilocybin or PCP act on serotonin receptors producing psychotic symptoms (Lieberman et al., 1998). Given the importance of alpha in visual processing, it is not surprising that most of the serotonin-induced alpha power modulations are described within the parieto-occipital regions. For example, Kometer et al. (2013) demonstrated a direct link between cortical excitability regulated by parieto-occipital alpha oscillations and visual hallucinations. To unveil the mechanistic role of 5-HT2_A_ receptors on the ongoing EEG activity, they designed a double-blind cross-over trial where a pre-treatment and a treatment were administered in the same experimental session. Participants were pre-treated with the antagonist ketanserin (50 mg) or placebo, and 1 h later, they could receive either the agonist psilocybin (215 μg/kg) or a placebo. Visual stimulation consisted of three “pacman” figures (300 ms duration) and the participants' task was to press a button as soon as they perceive the classical Kaniza illusion. Perceptually, psilocybin produced robust subjective alterations that were prevented by ketanserin pre-treatment. At the electrophysiological level, they found that psilocybin drastically reduced pre-stimulus alpha power (Kometer et al., 2013). These results support the notion that alpha oscillations regulate cortical excitability by pulsed inhibition, and the levels of alpha power could index visual gain (Klimesch et al., 2007; Mazaheri and Jensen, 2010). Nonetheless, the authors did not find a significant relationship between the drug-induced alpha suppression and the participant's reported intensity of visual hallucinations.

One of the most idiosyncratic alpha dynamics is its amplitude suppression after stimulus onset (Pfurtscheller and Lopes da Silva, 1999). Kometer et al. also investigated how serotonin upregulation impacted post-stimulus alpha power suppression and the results were very surprising. Psilocybin reduced the alpha power in such a way that there was no further power suppression after the stimulus (Kometer et al., 2013). Control analysis showed that the “absence” of alpha suppression was due to the strong psilocybin effects during pre-stimulus power periods. Importantly, the classical alpha dynamics were recovered using ketanserin as pre-treatment. This is a very important finding that establishes a causal link between alpha stimulus-induced power modulations and serotonin during visual processing. On the same note, the selective 5-HT2_A_ receptor activation reduced pre-stimulus alpha power to levels very close to “zero” (spectral power is positively-biased so spectral power can never be exactly “zero”). However, in line with (Muthukumaraswamy et al., 2013a), the psilocybin-induced alpha power suppression did not correlate with participants' visual hallucination rates. The lack of significant correlations between subjective scales and psilocybin-induced alpha power modulations could be due to floor/ceiling effects on subjective rating measures and/or alpha power levels.

In a very recent multimodal study, participants were scanned with functional magnetic resonance imaging (fMRI) and MEG during resting state conditions under the influence of LSD (Carhart-Harris et al., 2016). The psychedelic drug produced a noteworthy increase of cerebral blood flow over the primary visual cortex (as measured by fMRI). During MEG recordings, a robust suppression of parieto-occipital alpha power was found. Importantly, these LSD-induced brain activity changes the predicted quality and intensity of the visual hallucinations (Carhart-Harris et al., 2016). This study also found that LSD produced a remarkable increase in alpha frequency Figure 1d. What might be the functional relevance of this peak frequency change? LSD users often report dramatic visual disturbances or “visual trails” where they perceive a series of discrete stationary images trailing in the wake of otherwise normally moving objects (Dubois and VanRullen, 2011). To estimate the sampling rate of visual trails, Dubois and VanRullen created 10 short movies of 4 frames each, varying the inter-frame interval from 25 to 250 ms. They released the movies on a website and asked self-declared past LSD users to decide which movie best matched their recollection of psychedelic experiences. Interestingly, LSD users reported visual trail periodicities around 15–20 Hz (Dubois and VanRullen, 2011). Although highly speculative, the alpha power suppression and the frequency increase with LSD consumption might produce the segmentation of visual experiences into visual trails, a pharmacologically-induced form of perceptual cycles (VanRullen, 2016). New experiments are needed to test this possibility.

Finally, we still need to understand how serotonin interacts with posterior alpha and the consequences of this interaction for perception and cognition. First, the relationship between perception and alpha is well-documented (VanRullen et al., 2011; Lange et al., 2014). Second, there is growing evidence supporting the firing rate modulation as a function of alpha phase (Bollimunta et al., 2008; Lörincz et al., 2008, 2009; Haegens et al., 2011; Dougherty et al., 2017). Third, animal research has shown that serotonin regulates the power and frequency of neuronal oscillations (Celada et al., 2008; Puig et al., 2010); see (Puig and Gener, 2015) for a review). However, serotonin normalizes cortical excitability in a complex way. It is capable of producing either excitation or inhibition in interneurons and pyramidal cells, and even biphasic responses in the latter (Puig and Gulledge, 2011). How different cell types contribute to alpha oscillations through excitation, inhibition, or its balance and how this modulates behavior remains unknown. New studies are needed to address these issues.

## The next 10 years: computational modeling holds the key to advances in Pharmaco-MEEG

“*The art of modeling is about shaping a model to the questions that motivate it and then making highly educated guesses*” (Kopell, 2005).

Currently, we live in a research era where neurotechnological innovation is growing exponentially. New invasive recording techniques allow the simultaneous measurement of multiple brain regions, which in turn allow the inquiring of research questions that were impossible to address just a decade ago. However, this unprecedented amount of data acquisition raises serious challenges regarding data management, analysis, and interpretation. And this is where computational models could be crucial: “*The overarching goal of theory, modeling and statistics in neuroscience is to create an understanding of how the brain works—how information is encoded and processed by the dynamic activity of specific neural circuits, and how neural coding and processing lead to perception, emotion, cognition and behavior”* […]“*Coherent lessons must be drawn not only from the analysis of single experiments, but also by integrating insights across experiments, scales and systems. Theoretical studies will allow us to check the rigor and robustness of new conceptualizations and to identify distinctive predictions of competing ideas to help direct further experiments”* (Jorgenson et al., 2015). In line with Kopell's logic, I argue that pharmaco-MEEG needs modeling work in order to improve our mechanistic understanding of how different drugs affect brain activity, and to make *highly educated guesses* about how drug modulations drive (or hamper) cognition and behavior. In the paragraphs to follow, I briefly present examples of recent modeling work that has provided insights into how different neurotransmitters influence parieto-occipital alpha oscillations.

### GABA

Recent computational models account for the parieto-occipital alpha power decrease produced by GABAergic enhancers. A thalamo-cortical model has suggested two putative neural mechanisms that could explain this remarkable dipole position shift. The model proposes a Hodgkin-Huxley formalism where the frontal alpha power increase (and frequency decrease) can be explained by the effect of GABA imposing a temporal scale that entrains thalamo-cortical circuits (Vijayan et al., 2013). Simultaneously, the classical alpha power reduction may be caused by the effect of propofol hyperpolarizing the *h*-current channel on high-threshold (HT) thalamo-cortical cells, which are known to play a key role in the generation of posterior alpha rhythm (Lörincz et al., 2008, 2009). The *h*-current has been shown to be strongly involved in spindle generation (Destexhe and Babloyantz, 1993) but, as discussed earlier, any equivalence between alpha and spindles has to be taken with caution. This model is in line with the findings of an empirical fMRI-EEG study that shows that the pharmacological increase of GABA decreases both thalamic and posterior alpha activity (Schreckenberger et al., 2004).

Other models have highlighted that the interplay between inhibition and excitation might be a key feature of the alpha rhythm. Jones et al. (2000) developed a computational model generating alpha waves by the rebound excitation caused by GABAergic inhibition. They showed that if inhibition is increased pharmacologically, pyramidal cells fire less and alpha power (and frequency) decreases. Years later, Jones et al. (2009) further developed a cortical column model to reproduce the thalamo-cortical sensorimotor mu rhythm. They showed that the somatosensory mu rhythm appeared prominently when the feedforward thalamic input and the cortical feedback were in anti-phase (Jones et al., 2009). In this model, though, increasing GABA conductance in the feedforward 10 Hz component could lead to an increase of the cortical alpha power, which may explain the inverse relationship between firing rate and power found in some studies (Haegens et al., 2011; van Kerkoerle et al., 2014).

Another approach to modeling brain activity is the one proposed by neural mass models. Instead of modeling the properties of membrane potentials of hundreds of neurons, mass models describe the average activity of an underlying neuronal population (Robinson et al., 2001). The sum of different neuronal populations that can incorporate realistic biophysical parameters can describe macroscopic neural dynamics capable of reproducing and quantifying multiple EEG and MEG phenomena (Bojak and Liley, 2005). For example, (Robinson et al., 2001) modeled a thalamo-cortical loop where populations of excitatory and inhibitory neurons sent their activity to a thalamic population which was connected to the cortex through feedback loops. Hindriks and van Putten (2013) used this model to investigate how GABA modulates the resonance properties of the alpha rhythm. They suggest that the benzodiazepines decrease alpha power because their affinity for GABA_A_ receptors situated in pyramidal cells is higher when compared to inhibitory interneurons. The stronger action of GABA on principal cells decreases the firing rate to around 10 Hz, thereby, slowing-down the posterior alpha frequency. Taking recent empirical findings into account, the model is in line with the decrease of posterior alpha power with benzodiazepines, although the frequency modulation is less often reported. This might be due to the low dosages employed across studies.

### Acethylcholine and glutamate

A recent thalamic conductance-based model reproduced these experimental findings (Vijayan and Kopell, 2012). The model is a network constituted by RE cells, TC cells, HT cells, and interneurons. HT cells provide excitation to interneurons that inhibit TC cells. At the same time, HT and TC cells are connected to RE by AMPA receptors. Furthermore, RE cells inhibits HT cells, TC cells and their own local RE populations by GABAergic inhibition. The model postulates that the phase-shifting depends on mAChR (not on mGluR) and on the burst-firing mode of thalamic inhibitory interneurons. Interneuron single-spike firing favors the in-phase spike-alpha peak relationship and the bursting mode shifts the phase preference by π (Vijayan and Kopell, 2012). Moreover, the model makes several testable predictions. Cholinergic activity is very important to control the phasic modulation of the thalamo-cortical alpha rhythm that could lead to perceptual cycles (VanRullen, 2016). Low activation of excitatory mGluR still preserves the mAChR-induced alpha spike-field coherence, whereas strong mGluR activation disrupts it—silencing TC cells by RE and interneurons. Consequently, cortico-thalamic glutamatergic activation could induce an alpha component that prevents a sizable part of thalamic outputs from reaching the cortex. In summary, if the model reflects the machinery behind physiological alpha, mAChR should produce ~10 Hz oscillations important for stimulus processing while mGluR should generate a different alpha rhythm with a functionally inhibitory role (Vijayan and Kopell, 2012). This model is in line with the speculations I outlined above where alpha oscillations exerting *functional inhibition* could be implemented by *physiological excitation*.

#### 5-HT

Studies like Kometer et al. and Carhart-Harris et al. are amongst the first ones to unveil the neurophysiological mechanisms of visual hallucinations. Still, computational models using plausible biophysical parameters are urgently needed to explain how serotonin modulation produces alpha modulations. Among the exceptions, Muthukumaraswamy et al. (2013a) combined pharmaco-MEG data with dynamic causal modeling (DCM) (Moran et al., 2007) to study the mechanism by which psilocybin desynchronizes posterior cortices. DCM employs neural mass models as generative models to relate specific neural architectures to the EEG. An important advantage of this approach is that one can use Bayesian inversion using the real data to fit a series of mass models with their respective physiological parameters. Muthukumaraswamy et al. (2013a) found that psilocybin produced a robust decrease in alpha power around the parietal cortex during resting state conditions. To describe a potential neural circuit that explains this decrease in energy of the alpha signal, they built a canonical cortical microcircuit represented by neuronal subpopulations of four cell types: spiny stellate, superficial pyramidal, inhibitory interneuron, and deep pyramidal. The neural mass model could reproduce the psilocybin-induced alpha power suppression by the increase in the excitability of deep-layer pyramidal cells (Muthukumaraswamy et al., 2013a). The authors interpreted this finding within the predictive coding framework where the effect of feedback connections is believed to be inhibitory (Bastos et al., 2012). However, a seminal paper showed that feedback connections are not always inhibitory and stimulation within the classical or extra-classical receptive field can either excite or inhibit downstream regions (Hupé et al., 1998). It is hoped that future studies will test whether increasing the deep-layer pyramidal activity through serotonin 5-HT2_A_ activation yields a suppression of alpha power in posterior cortices.

## Methodological considerations for future studies

Traditionally, pharmacological studies have been carried out using EEG (Fink, 1969; Niedermeyer and da Silva, 2005), and more recently also MEG (Hall et al., 2010; Muthukumaraswamy, 2014). EEG and MEG measure electric and magnetic fields, respectively, associated to postsynaptic potentials generated by large pools of pyramidal neurons whose activity is coherent in time and space (Niedermeyer and da Silva, 2005). When presynaptic cells release excitatory neurotransmitters such as glutamate, they bind to ionotropic α-amino-3-hydroxy-5-methyl-4-isoxazolepropionic acid (AMPA) and N-methyl-D-aspartate (NMDA) receptors, producing excitatory postsynaptic potentials (EPSP). Conversely, inhibitory postsynaptic potentials (IPSP) are generated by gamma-aminobutyric acid (GABA) inhibitory receptors that hyperpolarize postsynaptic cells. These neurotransmitters mediate excitatory and inhibitory intracellular primary currents (MEG sensitive) which are compensated by balanced extracellular return currents (EEG sensitive). While single neuron action potentials (neural output) produce large currents that are too brief and asynchronous to be temporally integrated, postsynaptic potentials (neural input), in contrast, have a half-life an order of magnitude longer (Timofeev et al., 2012), thereby widening the critical window of temporal integration. In the same vein, spatial integration is archived due to pyramidal cells whose axons are aligned in parallel, producing the so called “open field” configuration (Lorente de Nó, 1947). If a sufficient number of excitatory cells fire in synchrony (temporal integration), the open field configuration (spatial integration) can produce a measurable net potential that extends throughout the conducting medium. In clear contrast, the morphology of inhibitory interneurons is shaped radially, yielding sinks and sources that cancel each other, contributing (in principle) little to EEG and MEG signals (Murakami and Okada, 2006). In sum, the alpha waves recorded with EEG and MEG are mainly driven by spatio-temporally coordinated post-synaptic currents generated by pyramidal cells.

MEG and EEG pharmacological trials involve higher complexity during data acquisition and analysis than standard experiments. In this section, I will highlight two of these challenges and I will suggest possible ways to approach them. For an excellent methodological review about pharmaco-MEG, the reader is referred to Muthukumaraswamy (2014).

### The role of common denominator when comparing normalized power differences

The binding of pharmacological agents to pyramidal cells and interneurons can produce dramatic changes in EEG and MEG activity. It is thus important to investigate the brain region/s where specific electrophysiological changes seem to be caused by the pharmacological intervention, discarding non-specific changes that covary with the treatment effect but are not related to it. Absolute spectral power estimates are very popular dependent measures routinely reported in pharmacological trials. However, this index can suffer from large variations across experimental sessions, participants, or drug treatments. To facilitate comparisons across studies (or across trials, participants, sessions, etc.) it is highly recommended to employ frequency specific power normalization. Often, this normalization is performed by comparing a specific time window (i.e., post-stimulus) with a baseline period; or, by comparing various task-relevant time windows (i.e., left vs. right allocation of attention). Sometimes drug power or amplitude modulations can be frequency and time specific, that is, only a specific band or time segment can be affected by a drug. For example, Kometer et al. (2013) demonstrated that the impact of psilocybin on alpha power was stronger during the pre-stimulus period, with non-significant post-stimulus drug modulations. In these situations, the computation of a relative power change between post- and pre-stimulus periods can “move” the significant differences toward the post-stimulus period.

**Figure 2:**
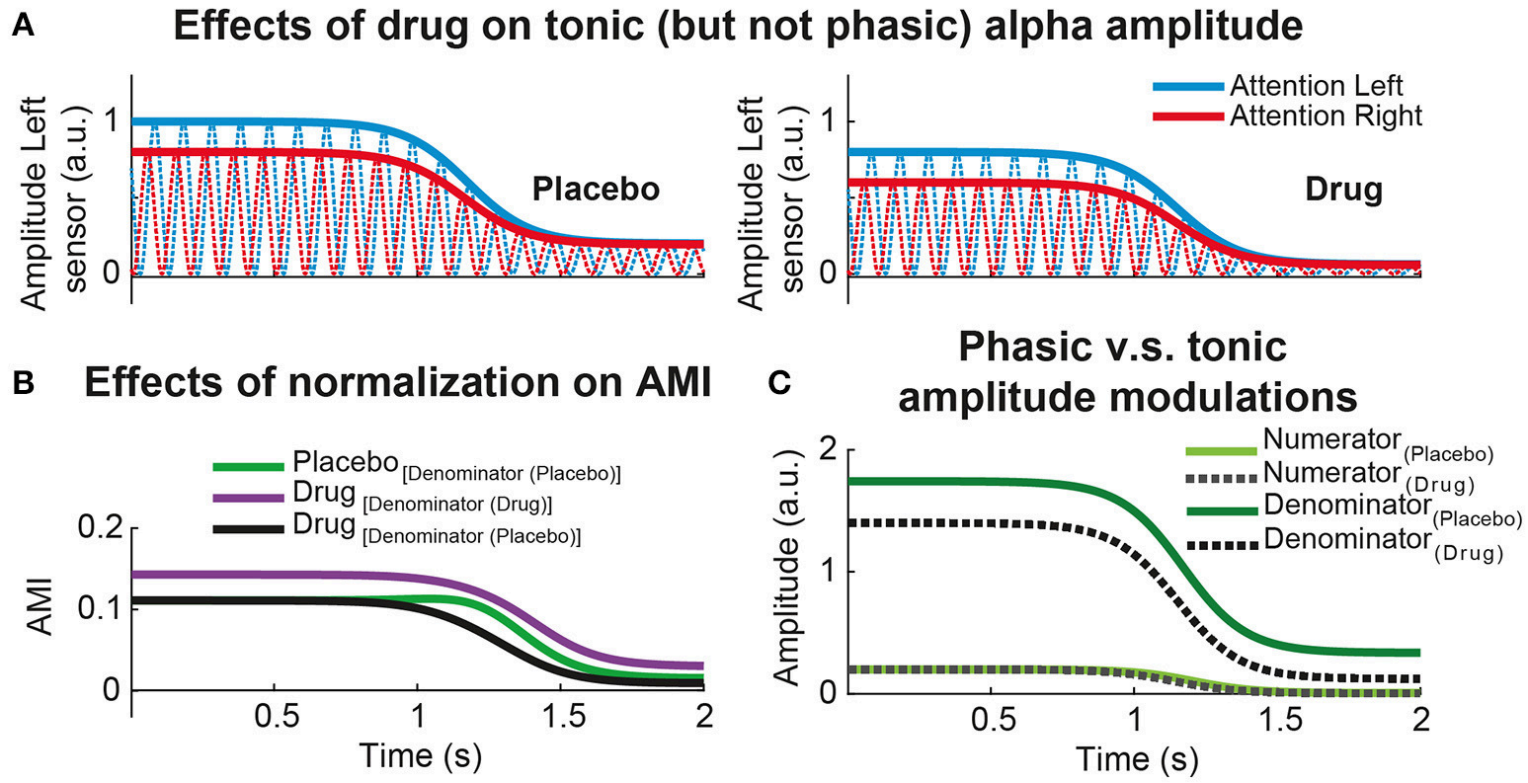
Effect of common denominator when comparing normalized power differences. **(A)** Simulated alpha power dynamics of a hypothetical MEG sensor from left hemisphere in a visuo-spatial attention task. Red (blue) lines represent the alpha oscillations during attention to the right (left) conditions. The left column illustrated the alpha attentional power modulations during Placebo and the right column during an experimental session where a hypothetical Drug exclusively produces a tonic power reduction ànd leaves the attentional power modulation intact. The power differences between attention to the left (blue) vs. attention to the right conditions (red) is the same for Placebo and Drug. **(B)** Time-resolved alpha modulation index (AMI) for Placebo and Drug sessions. AMI is defined as the normalized power difference between attentional conditions: (attention to the left – attention to the right)./ (attention to the left + attention to the right). AMI is compared between treatments using different denominator (green vs. purple) or using the same, in this case Placebo (green vs. black). **(C)** Time-resolved power differences due to attentional (phasic) alpha power modulations (numerator) vs. total (tonic) alpha power (denominator) for each treatment separately. In this simulated example, it is clear that the Drug decreased the total or tonic alpha power (denominator) without interacting with attention (numerator). As the tonic power during Drug is lower relative to Placebo, having the same attentional modulation, the AMI is clearly enhanced using different denominator relative to common denominator (see **B**).

Along the same lines, the use of normalized oscillatory power difference in a pharmaco-MEEG study needs special attention. Alpha modulation index (AMI) and alpha lateralization index (ALI) are very popular metrics to quantify how oscillatory power or amplitude changes as a function of allocation of attention (Thut et al., 2006; Händel et al., 2010). Within the context of a standard spatial attention task, AMI is defined as the alpha power or amplitude difference between the experimental trials where participant's attention is cued to the left and the right experimental conditions: AMI = (Attention Left – Attention Right)./ (Attention Left + Attention Right). In principle, a drug can either increase or decrease the tonic power of the alpha rhythm. Consequently, and under some specific conditions, the denominator of the AMI index can significantly differ between the two treatment conditions (placebo vs. drug). To understand the specific (or general) effects of a drug, it is advisable to compare absolute and relative power or amplitude differences. It may be the case that the numerator of AMI has very similar amplitude or power values for placebo and treatment (although power is the squared amplitude, the rationale of the following example will remain qualitatively the same for both measures). This is indeed the case displayed in Figure 2A: the phasic alpha amplitude modulation (the one associated to the allocation of attention) is almost identical for placebo and drug sessions. If the drug strongly modulates tonic alpha amplitude (increasing or decreasing it), AMI might statistically differ due to tonic amplitude differences (Figure 2B) but not due to an interaction between the treatment and the amplitude modulations caused by participants' allocation of attention. More specifically, Figure 2C clearly shows how the increase in AMI during the drug session (Figure 2B) is explained by the differences in the denominators across treatments and not due to differences in the numerators). The same can happen to ALI, which is defined as the alpha power or amplitude contrast between the two hemispheres for a given spatial attention condition (Thut et al., 2006). To avoid this issue, it is recommended to use a common denominator by either taking the mean of alpha power (or amplitude) estimates over treatment conditions, or by using the estimates of the placebo session as a common reference (Figure 2B, black line vs. green line). In this case, it is possible to dissociate the influence of the drug on alpha amplitude modulations due to the allocation of attention (indexed by AMI) and the influence of the drug on tonic alpha amplitude (Figures 2B,C). In the simulated example depicted in Figure 2A, magnitude of the attentional alpha amplitude modulation was almost identical for Placebo and Drug sessions. The Drug, nonetheless, exclusively affected the level of tonic alpha amplitude (Figure 2C, denominator). Importantly, the same rationale can apply to any other type of intervention such as brain stimulation (transcranial and electric stimulation techniques), or when comparing normalized amplitude or power differences extracted from multiple experimental conditions.

### Minimizing head movements during MEG experiments

Head movements during MEG recordings are a common source of noise that the experimenter needs to control as much as possible. In EEG set-ups, the electrodes are attached to the scalp of the participant and small head movements can easily be reduced using chin-rests. In a MEG set-up the problem is the other way around: the participant fits his/her head to the helmet, trying to stay and still as possible. In supine position, it is relatively easy for the participants to remain still. But when sitting down on the MEG chair, head position can drift during the experiment, especially during long sessions (>30 min). Taking this into account, choosing the supine position is, in principle, a natural choice; but the physiological results may drastically differ relative to sitting position (Rice et al., 2013). Within the context of pharmaco-MEG, participants may move their heads differently during the treatment session due to the side effects of the drugs. Therefore, signal differences between placebo and drug sessions could partially be explained by the difference in head position in the two conditions, thereby limiting the interpretability of the results. It is well-known that dipolar electromagnetic fields decrease at a rate of 1/r^2^ with the distance of the coil (Hämäläinen et al., 1993), so drifting down on the MEG chair can reduce the amplitude of the MEG signal considerably. In addition, multiple head movements along different directions will contribute to a decrease in the signal-to-noise ratio of the MEG signal. This in turn may eventually diminish the statistical sensitivity of the experimental manipulation at hand. Source reconstruction methods can take into account the distance from participants' head relative to the MEG coils on a trial-by-trial basis, or averaging the head position during the whole experiment (Stolk et al., 2013). Unfortunately, this approach cannot solve all possible caveats. For example, if the head position of a participant lies at the back of the dewar during the first half of the experiment, and then moves a few centimeters to the right (left) or to the front of the dewar, source reconstructions can yield very different results when comparing the first and the second half of the experiment.

In general, there are two ways to minimize head movement: during the online acquisition of the data and during the post-processing of the MEG data. The latter procedure employs, for example, an interpolation of the magnetic field distribution produced by the inversion of a forward model generated by taking into account the actual head movements (Knösche, 2002). A different sensor interpolation approach is employed during the signal space separation, where a set of spherical harmonic functions are able to separate the influence of signals originating outside of the MEG coils and, simultaneously, separate artifacts close to the coils (i.e., head movements; Taulu et al., 2005; Medvedovsky et al., 2007). Unfortunately, the MEG signal may be drastically affected when correcting for large head movements (Gross et al., 2013). Taking all these factors into account, it is highly desirable to record head position and to minimize position changes as much as possible during the online acquisition of the experimental data.

**Figure 3:**
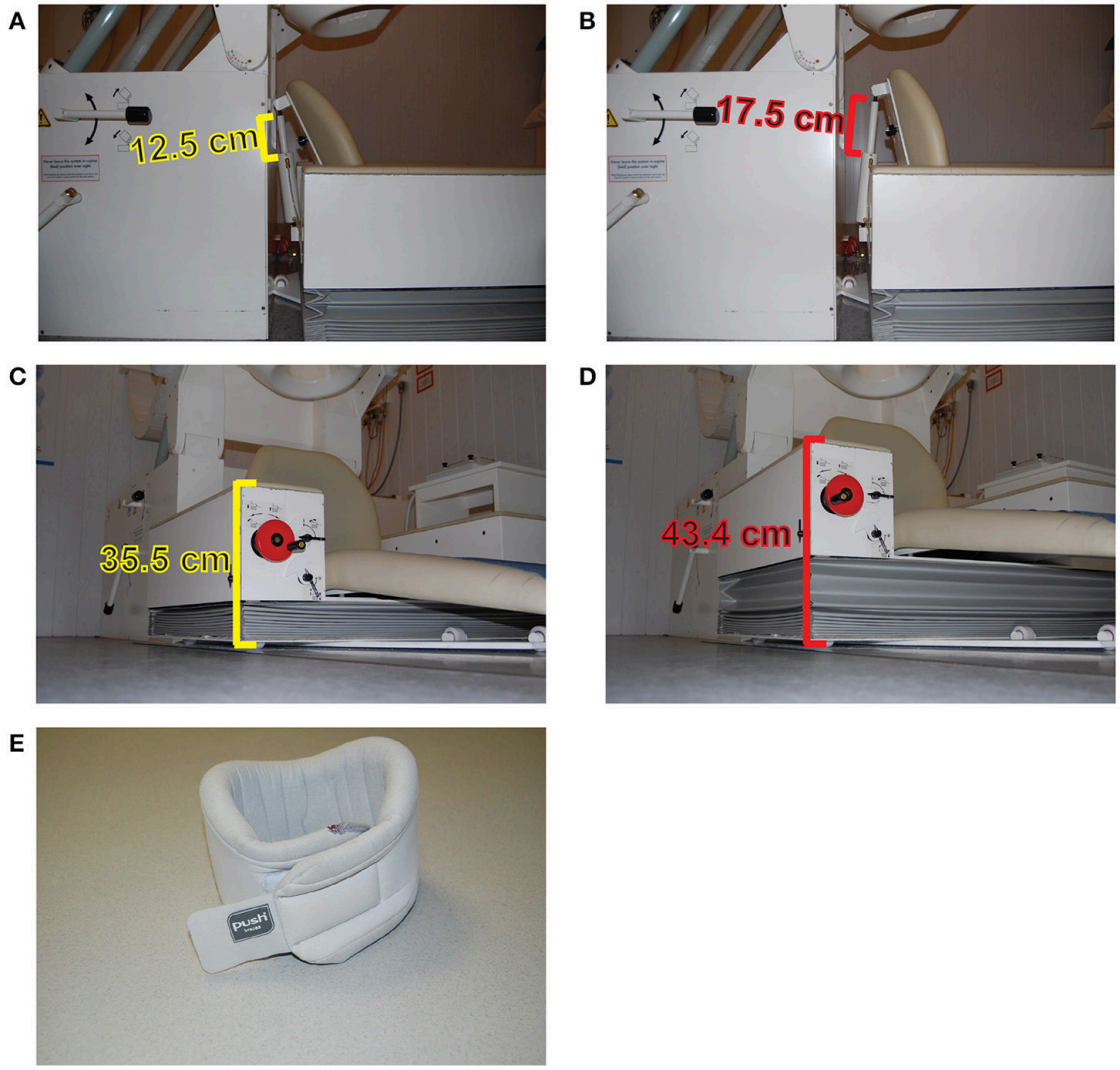
Minimizing head movements during MEG experiments. Measurement of the MEG chair inclination of the CTF275 system in two specific positions **(A,B)**. The height of the chair in two different positions **(C,D)**. Example of a sponge neck-collar **(E)**.

A simple and cheap solution is the use of orthopedic neck-collars combined with chair measurements (Lozano-Soldevilla et al., 2014). Especially for MEG experiments involving multiple experimental sessions separated by days, it is very important (and difficult) to seat the participants in the same position. A simple solution consists of taking the height and inclination measurements from the MEG chair for each participant and to reproduce the chair position during subsequent experimental sessions (Figures 3A–D). In addition, soft sponge type neck-collars can easily adapt to most participants while keeping them relatively comfortable. When attached to the neck, one can instruct the volunteers to rest his/her chin on the top of the collar (Figure 3). This action releases the neck muscles from holding the weight of the whole head and reduces fatigue. This approach was especially beneficial because the nature of the drug manipulation in our study. We used benzodiazepine lorazepam in two different dosages to increase GABA efficacy (Lozano-Soldevilla et al., 2014). Lorazepam produces loss of muscle tone, so muscles relax and suffer fatigue faster relative to placebo. The use of the collar reduced the fatigue of neck muscle while keeping participants relatively comfortable. That said, besides evidence from their own work, the author is not aware of any empirical measurement demonstrating the superiority of this approach relative to other procedures. However, many researchers carrying out MEG experiments at the Donders Institute have employed them the in the past (Grent-'t-Jong et al., 2013, 2014; Marshall et al., 2016; van de Nieuwenhuijzen et al., 2016), and still continue to employ them (Drijvers et al., 2018) due to good feedback from participants.

A more advanced procedure to monitor and correct head movements was provided by Stolk et al. (2013). They created a real-time head-localizer tool, which is a part of the FieldTrip Matlab toolbox (Oostenveld et al., 2011). For example, in the CTF275 MEG system, head coil positions can be placed at anatomical landmarks (nasion, left and right ear canals) to record head movements. At the beginning of an MEG experiment, head position can be tracked and stored. In case of excessive head movement (i.e., > 5 mm), the experimenter can stop the task and instruct the participant to correct his/her head position to recover the original coordinates. Very often, pharmaco-MEG studies employ cross-over designs: the same participant receives placebo and treatment/s in different sessions separated by days. The real-time head-localizer tool allows the experimenter to recover the tracked positions acquired in the very first session so that they can guide the participant to sit as close as possible to the original position (Lozano-Soldevilla et al., 2014).

Finally, it is likely that the future of the MEG and pharmaco-MEG research will involve 3D printed head-casts which will drastically improve the online acquisition of data upon the present situation. Initial measurements have revealed impressive results with within- and between-session head movements below 0.25 and 1 mm, respectively (Meyer et al., 2017).

## Limitations

Psychopharmacological research is essential to monitoring electrophysiological effects of new drugs, and to compare them with well-established treatments. The employment of drugs with well-characterized mechanisms of action can help to develop translational research programs thanks to the priceless knowledge gained in animal models. At the same time, the progress in animal and human psychopharmacological research is very important to constrain computational models with biophysically realistic parameters. These models, once contrasted with empirical data, can help to guide future research proposing mechanistic hypothesis about how specific synaptic receptors generate and/or modulate neuronal oscillations at various frequencies.

However, as with any other approach, pharmaco-MEEG is not exempt from limitations. One of the most important limitations is that pharmacological interventions are system-wide. Moreover, drug agents rarely target one receptor type and will bind to any brain region that contains those specific receptors. It is also challenging to select an optimal drug dosage to produce a measurable behavioral effect not explained by global side effects. Despite the difficulties, there are well-designed studies showing drug-specific effects. For example, Hall et al. (2010) were able to untangle the diazepam-induced resting state neuronal networks into different frequency bands. In a visuospatial attention task, physostigmine strongly enhanced frontal gamma power leaving the stimulus-induced visual gamma “virtually intact” (Bauer et al., 2012). These results point toward a promising future for pharmaco-MEG studies (Hall et al., 2010; Muthukumaraswamy, 2014) and the future development of pharmacological models which test the mechanistic role of neuronal oscillations.

Another important limitation consists of the uncertainty in the ecological validity of the pharmacological model. For example, it has been recently suggested that neuronal oscillations generated with sedative drugs and anesthetics are neurophysiologically different from natural sleep (Akeju and Brown, 2017) despite the striking similarity of some brain states, like the slow waves.

Other main limitation of the pharmacological study of alpha oscillations is that we still do not know the main sources and circuitry of this brain rhythm. Without this knowledge, it is difficult to build *in-vitro* and computational models (see Hughes and Crunelli, 2005 for an exception). The alpha rhythm seems to be a network-based oscillation with multiple local sources interacting each other. In contrast, faster rhythms seem to strongly depend on local circuitry which has facilitated its study in both animal and computational models (Traub et al., 1999). The alpha rhythm can be found over multiple brain regions, even during resting state conditions (Hindriks et al., in review). In addition, using new decomposition techniques, it has recently been found that both attention and performance can be positively and negatively correlated with multiple overlapping alpha and beta sources located over task-relevant and task irrelevant brain regions (van der Meij et al., 2016). This is in line with the disparate pharmacological modulations reviewed here. These two findings reveal that there are multiple alpha components performing complementary roles. How each alpha component is affected by different pharmacological compounds modulating behavior remains to be elucidated. To conclude, these observations are not new and were eloquently described by W. Gray Walter a long time ago:

“*We've managed to check the alpha band rhythm with intracerebral electrodes in the occipital-parietal cortex; in regions which are practically adjacent and almost congruent one finds a variety of alpha rhythms, some of which are blocked by opening and closing the eyes, some are not, some are driven by flicker, some are not, some respond in some way to mental activity, some do not. What one sees on the scalp is a spatial average of a large number of components, and whether you see an alpha rhythm of a particular type or not depends upon which component happens to be the most highly synchronized process over the largest superficial area; there are complex rhythms in everybody*.” (Pfurtscheller et al., 1994).

In the future, it will become important to combine computational modeling with pharmacological work to improve the mechanistic understanding of how different receptors contribute to the modulation of posterior alpha oscillations. This knowledge will help the community to develop translational research programs to investigate the pathophysiology of brain rhythms in diseases with well-characterized electrophysiological abnormalities such us epilepsy, Parkinson disease, schizophrenia, or cerebellar ataxia (Traub and Whittington, 2010).

## Outstanding questions

The models and experimental findings I have reviewed above provided invaluable insights. Nonetheless, posterior alpha oscillations still contain many secrets that future research, hopefully, will unveil:

- It is necessary to continue investigating the sources of parieto-occipital alpha oscillations. While the thalamic mechanisms have been relatively well-studied both empirically (Hughes and Crunelli, 2005; Lörincz et al., 2008, 2009) and computationally (Vijayan and Kopell, 2012), the cortical alpha mechanisms are less clear. To improve the mechanistic understanding of the posterior alpha, *in-vitro* preparations will be fundamental (Florez et al., 2015) to generating realistic computational models.
- What is the specific contribution of inhibitory interneurons and pyramidal cells to the modulation of alpha frequency and power? Like other inhibitory rhythms such as gamma, can alpha oscillations be exclusively generated by interneurons (Whittington et al., 1995)?
- On a short time scale, alpha frequency is known to fluctuate, and this modulation has been related to the latency of visual P1 (Himmelstoss et al., 2015). In rat hippocampus, it has been shown that instantaneous gamma frequency and power are determined by the tight balance between excitation and inhibition (Atallah and Scanziani, 2009). Is there a similar physiological mechanism to control alpha power and frequency?
- A new impressive study has characterized the beta waves as non-periodic events with a non-sinusoidal waveform caused by the synchrony of excitatory input currents to cortical pyramidal neurons (Sherman et al., 2016). It is well-documented that there is an asymmetry between the magnitude of the peaks and troughs of posterior alpha oscillations (Stam et al., 1999; Nikulin et al., 2007; Mazaheri and Jensen, 2008). Can we extract wave morphology metrics to make inferences underlying the physiology of parieto-occipital alpha oscillations (Cole and Voytek, 2017)? What are the key neurotransmitter(s) and cell types that control alpha waveform and what is its functional relevance (if any)?
- Some studies have shown a relationship between the phase of visual alpha oscillations and neuronal firing (Bollimunta et al., 2008, 2011). Moreover, some studies report the relationship between alpha power and spiking activity as being inverse (Nir et al., 2007; Haegens et al., 2011; van Kerkoerle et al., 2014), some report a positive correlation (Mo et al., 2011; Lakatos et al., 2016), and some studies report no apparent relationship at all (Dougherty et al., 2017). What is the reason for these diverse findings? Is it related to the action of neuromodulators such serotonin or acetylcholine?
- There are only few studies that have found an association between dopamine and posterior alpha oscillations (Chapotot et al., 2003; Eckart et al., 2014) but see (Bodenmann et al., 2009; Albrecht et al., 2016) for negative results. Given the well-established role of dopamine in cognitive control and working memory (Cools and D'Esposito, 2011), and the modulation of posterior alpha power during top-down operations (van Kerkoerle et al., 2014; Bastos et al., 2015; Michalareas et al., 2016), it would be interesting to study how dopamine levels interact with posterior alpha during capacity-limited working memory conditions. Specifically, it has been shown that the relationship between dopamine levels and working memory performance is described by an inverted-U shaped curve. For instance, one could predict that participants with low baseline working memory capacity may increase their performance using dopamine enhancers (Cools and D'Esposito, 2011). It remains to be tested whether posterior alpha desynchronization during the delay interval (Fukuda et al., 2015) interacts with dopamine levels.
- It is still unclear why posterior alpha power dynamics are modulated as a function of working memory contents. Using a Sternberg task employing letters as memoranda, (Jensen et al., 2002) convincingly showed that alpha power during delay interval parametrically increased with memory load. Strikingly, the opposite result is consistently reported using visuospatial working memory tasks (Fukuda et al., 2015). A previous working memory fMRI study demonstrated that physostigmine induced stronger activity in ventral stream relative to dorsal (Handjaras et al., 2013). Could it be that the increase in alpha power in working memory tasks using letters as memoranda is a consequence of the cholinergic neuromodulation of the ventral stream?

## Conclusion

In conclusion, recent human pharmacological studies have investigated how the main neurotransmitters and neuromodulators in the central nervous system impact the posterior alpha rhythm. In general, the alpha power was found to decrease with GABAergic enhancers, glutamate blockers and serotonin enhancers, whereas an increase was often reported with acetylcholine. Currently, there is no theoretical framework that can explain these pharmacological findings. Based on these studies, I speculate that the physiological basis of the classical alpha oscillations could depend on *physiological excitation* to a similar (or stronger) extent as *physiological inhibition*. Considering the complexity underlying the alpha rhythm, future studies are needed to address how physiological excitation and inhibition contribute to generate the parieto-occipital alpha oscillations. I speculate that a new generation of computational models will be crucial to a mechanistic understanding of alpha oscillations and to reproduce the variety of empirical findings. “*How does one draw a conclusion from such a model? It is almost universally believed that with enough parameters in a model, one can reproduce anything. But it is actually the inability to reproduce some details, while trying as hard as possible to be “faithful,” that produces the key clues. (The successful use of this methodology requires intense and on-going self-criticism.)”* (Kopell, 2005).

## Author contributions

DL-S analyzed data; interpreted results of experiments; prepared figures; drafted manuscript; edited and revised manuscript; approved final version of manuscript.

### Conflict of interest statement

The author declares that the research was conducted in the absence of any commercial or financial relationships that could be construed as a potential conflict of interest. The reviewer XL and handling Editor declared their shared affiliation.
